# Anthelmintic treatment alters the parasite community in a wild mouse host

**DOI:** 10.1098/rsbl.2013.0205

**Published:** 2013-08-23

**Authors:** Amy B. Pedersen, Janis Antonovics

**Affiliations:** 1 Department of Biology, University of Virginia, Charlottesville, VA 22904, USA; 2 Centre for Immunity, Infection and Evolution, School of Biological Sciences, University of Edinburgh, Edinburgh EH9 3JT, UK

**Keywords:** helminths, community ecology, within-host interactions

## Abstract

Individuals are often co-infected with several parasite species, yet the consequences of drug treatment on the dynamics of parasite communities in wild populations have rarely been measured. Here, we experimentally reduced nematode infection in a wild mouse population and measured the effects on other non-target parasites. A single oral dose of the anthelmintic, ivermectin, significantly reduced nematode infection, but resulted in a reciprocal increase in other gastrointestinal parasites, specifically coccidial protozoans and cestodes. These results highlight the possibility that drug therapy may have unintended consequences for non-target parasites and that host–parasite dynamics cannot always be fully understood in the framework of single host–parasite interactions.

## 1. Introduction

Infectious diseases play a key role in the dynamics and regulation of wild populations through negative effects on host survival and fecundity [1]. It is becoming clear that individuals are often simultaneously co-infected with multiple parasite species and that those parasites may interact within a host [2–9]. This dynamic and complex parasite community may be structured either via direct interactions or indirectly through shared resources or immune responses [4,5]. From an applied perspective, understanding the role of such parasite interactions in shaping disease dynamics is crucial for optimizing intervention strategies.

To date, most evidence of interspecific parasite interactions from wild populations has been observational and based on cross-sectional or longitudinal surveys which correlate parasite infections to infer interactions [3,10]. However, as is well known in community ecology, only a more direct, experimental approach can ascertain the consequences of these interactions for disease dynamics [7].

In this study, we investigate the effects of removing one taxonomic group of parasites on the prevalence and intensity of other non-target parasite species, as well as on host recapture rates under natural conditions. We experimentally reduced nematode infections in wild populations of *Peromyscus leucopus* (white-footed mouse) and *Peromyscus maniculatus* (deer mouse) and monitored the intestinal parasite and ectoparasite community. We show that nematodes may exert a strong, yet unexpected, antagonistic force on non-target parasites, with important potential consequences for parasite community composition, dynamics and host health.

## 2. Material and methods

This study was conducted in an oak–maple forest at Mountain Lake Biological Station (MLBS) in Virginia, where populations of *Peromyscus* have been studied for more than 25 years [11,12]. In this locality, *Peromyscus* species share more than 10 species of intestinal parasites (including *Aspicularis americana*, *Capillaria americana*, *Syphacia peromysci*, *Eimeria* spp., *Hymenolepis dimunata* and *H. citelli*), several ectoparasites (including ticks, fleas and *Cutebra* sp.) and co-infection is common ([13] and electronic supplementary material, table S1).

Six 0.5 ha trapping grids (8 × 8 arrays with 10 m spacing) were established and mice were trapped for three consecutive nights every two weeks in June–August 2003. Sherman folding traps were set at dusk, baited with crimped oats and checked the following morning. Grids were separated by more than 70 m. In four randomly selected ‘experimental’ grids, individuals at first capture were randomly assigned to receive either a single oral dose of ivermectin (200 μg kg^−1^) or an equal volume of water. Ivermectin removes nematode infections and some ectoparasites, yet has no known negative effects on host fitness [9,12]. In two ‘control’ grids, all individuals were given water.

At each capture, individuals were ear-tagged and their species, sex, age, weight, length and reproductive condition were recorded. *Peromyscus maniculatus* was distinguished from *P. leucopus* based on tail length exceeding body length, sharply bicoloured tail and a hair tuft at the end of the tail. Developmental age (juvenile, subadult and adult) was determined by pelage colour. Males with testes greater than 6 × 4 mm, and females with a perforate vagina, lactating nipples or who were pregnant, were considered to be reproductive.

*Peromyscus* abundance was measured as the minimum number known alive by summing the individuals caught in the session with those trapped at prior and later sessions. Faecal samples were taken from every capture, and individuals were scanned for ectoparasites (presence/absence). All traps were then cleaned and sterilized with a hospital-grade detergent.

Faecal samples were weighed and stored in 10 per cent buffered formalin at 4°C. All samples were analysed within four months of capture. Salt flotations and microscopy were used to count eggs/oocysts [14]. Two measures were used to describe intestinal parasite infection: (i) prevalence and (ii) intensity of infection (eggs/oocysts per gram faeces), a common proxy for worm burden [15].

We analysed the effect of ivermectin on target and non-target parasites using generalized linear models (GLMs) and generalized linear mixed models (GLMMS) in R v. 2.13 [16]. We tested fixed effects: time, treatment, grid, date of first capture, age, sex, species, interactions and importantly treatment × time. Mouse ID was included as a random effect to control for multiple samples per mouse over time. GLMs and GLMMs gave very similar results, likely due to low captures/mouse (mean = 1.7). Conclusions were unaffected by model type. GLMs are usually more robust, especially for non-normal response variables and unbalanced data [17], thus the GLMMs are presented in the electronic supplementary material. For prevalence, we used a binomial error structure, and for parasite intensity (log-transformed as the data were eggs per gram of faeces), we used a Gaussian error structure. Full models were simplified by backward stepwise elimination of non-significant terms (*p* > 0.1) to obtain the minimum adequate model. To measure the effect of ivermectin treatment on survival (weeks recaptured post-treatment), we used a GLM, with Poisson error structure and the fixed effects listed above, and capture–mark–recapture methods to measure survival and recapture probabilities in the program Mark [18].

Data will be made available after a 1 year embargo. Until this time, data are available upon request from the authors.

## 3. Results

During the experiment, 270 individuals were tagged and treated (90 with water on control grids; 88 were treated with ivermectin and 86 with water on experimental grids, totalling 453 captures (see the electronic supplementary material, figure S1 for demographic patterns). There were no significant differences in species composition, sex or parasite prevalence of untreated animals between control and experimental grids; these data were therefore combined for further analyses.

### (a) Effects on target and non-target parasites

Ten intestinal parasite species were identified to taxonomic class: coccidia (three spp.), nematodes (five spp.) and cestodes (two spp.); as well as three ectoparasites (see the electronic supplementary material, table S1); because not all parasites could be identified to species, analyses were at the class level.

**Figure 1.**
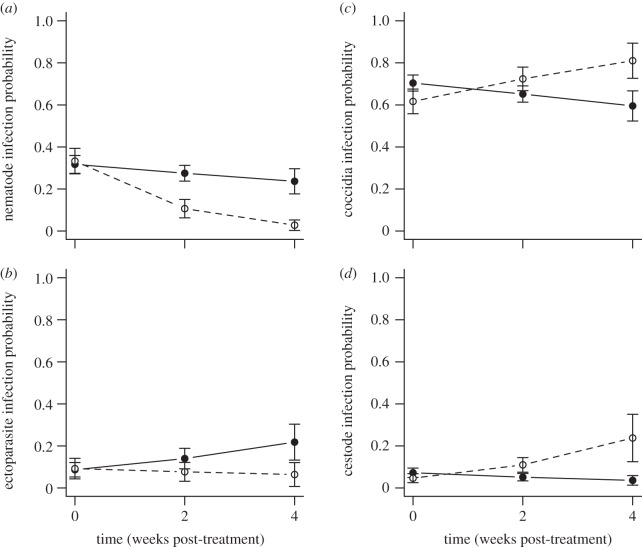
The effect of ivermectin on the probability of infection of drug-target parasites: (*a*) nematodes and (*b*) ectoparasites; and non-target parasites: (*c*) coccidial protozoans and (*d*) cestodes of ivermectin-treated (dashed line; open circles) and control (water; solid line; filled circles) mice from the GLM models (table 1). Week 0 represents pre-treatment infection probability, and week 2 and 4 represent recaptured individuals after treatment. Bars represent s.e.

Ivermectin treatment reduced the prevalence of intestinal nematodes by more than 28 per cent; whereas in control mice prevalence decreased by only 8 per cent (treatment × time: *F*_1,390_ = 6.42, *p* = 0.0112; table 1 and figure 1*a*). Treated individuals tended to have lower ectoparasite prevalence than controls, although this was not significant (treatment × time: *F*_1,390_ = 2.18, *p* = 0.13; table 1 and figure 1*b*). Prevalence of ectoparasites (primarily driven by botfly infections) increased by more than 15 per cent in controls, but was unchanged in ivermectin-treated mice.

**Table 1.** Results from the minimum adequate binomial GLMs on the change in parasite prevalence post-ivermectin treatment. Statistics show the chi-squared value, d.f. and *p*-value, with *n* = 397. The treatment × time interaction is the test of the experimental effect of ivermectin (italicized). Models are plotted in figure 1.

	nematode prevalence	ectoparasite prevalence	coccidia prevalence	cestode prevalence
grid	—	9.86_5_ (0.08)	—	—
first capture	—	*13.2_2_* (*0.001*)	—	—
age	*4.17_1_* (*0.041*)	—	—	2.0_1_ (0.16)
sex	*4.189_1_* (*0.041*)	—	2.52_1_ (0.11)	1.48_1_ (0.22)
species	1.68_1_ (0.195)	—	*6.8_1_* (*0.009*)	—
treatment × time	*6.42_1_* (*0.0112*)	2.18_1_ (0.13)	*4.17_1_* (*0.04*)	*5.49_1_* (*0.02*)

Ivermectin-treated mice had a significantly higher prevalence of coccidia than control mice (treatment × time: *F*_1,390_ = 4.17, *p* = 0.04; table 1 and figure 1*c*), increasing by more than 20 per cent four-weeks post-treatment, while decreasing in control individuals by 10 per cent over the same period. Cestode prevalence also significantly increased by approximately 20 per cent in ivermectin-treated mice, whereas prevalence remained relatively constant in controls (treatment × time: *F*_1,390_ = 5.49, *p* = 0.02; table 1 and figure 1*d*).

Among untreated nematode-infected individuals, the average infection intensity was 226.6 eggs per gram (see the electronic supplementary material, table S1). Ivermectin treatment did not significantly reduce nematode intensity, however all treated-mice were uninfected by four-weeks. There was also no significant effect of ivermectin treatment on non-target parasite intensity.

### (b) Effects on host fitness

The encounter rates for control and ivermectin-treated mice were more than 95 per cent. Ivermectin-treated mice were recaptured for, on average, 1.35 weeks after treatment, whereas control mice were recaptured for 1.57 weeks; this difference was not significant (*p* > 0.1; see the electronic supplementary material for more details). Ivermectin-treated or control groups did not differ significantly in the proportion of reproductive male or female mice post-treatment.

## 4. Discussion

These results suggest the presence of antagonistic interspecific parasite interactions in a natural population of mice. Removing one group of intestinal parasites had significant, and unexpected, effects on the parasite community within an individual. Ivermectin treatment successfully decreased intestinal nematode infection, but this was accompanied by increases in non-target coccidia and cestodes. The mechanisms driving these interactions are currently unknown. They may be due to either a ‘bottom-up’ process (via competition for space/resources) because all parasite groups inhabit the gastrointestinal tract or a ‘top-down’ (via shared immune responses) interaction, owing to immune-mediated mechanisms such as enterocyte turnover, which is a host response elicited by helminth infection that can reduce resources available to coccidia [18]. Nematode and coccidia infection, but not intensity, were affected by ivermectin treatment, which may suggest that the interaction affects parasite establishment and not within-host replication. In addition, it is possible that ivermectin treatment increased exposure to non-target parasites via changes in behaviour or dietary habits; further experiments are needed to determine the specific mechanisms.

Quite unexpectedly, there was no evidence that reducing nematode infection increased the fitness of treated mice. In fact, ivermectin-treated mice had lower recapture rates than control mice. Although this effect was not significant, our results are counter to studies showing increased fitness following removal of a single dominant nematode parasite [12,19,20]. In this study, ivermectin treatment increased the prevalence of non-target gastrointestinal parasites, especially coccidia, which have been associated with decreased mass and lower over-wintering survival in *P. maniculatus* [21] and with high juvenile mortality in other mammals [22]. We found no difference in reproductive condition between treated and control individuals. However, a larger-scale experiment that targets different parasite groups and tracks the longer-term host–parasite dynamics would provide a clearer picture of the network of interspecific interactions within the parasite community and their consequences for host health.

In conclusion, we find that interspecific parasite interactions can be assessed through a field-experimental approach, rather than through classical indirect observational studies using parasite infection data. It is clear that parasite–host dynamics cannot always be understood within a single-host–single-parasite framework. Like ecological communities of free-living species, parasite communities are dynamic and structured by interactions that determine species presence/absence and intensity. This is consistent with the increasingly accepted view that parasite control strategies need to take parasite community structure into account for effective disease management of human and animal diseases [23].
